# Improvement of Diabetes Mellitus After Colorectal Cancer Surgery: A Retrospective Study of Predictive Factors For Type 2 Diabetes Mellitus Remission and Overall Survival

**DOI:** 10.3389/fonc.2021.694997

**Published:** 2021-07-06

**Authors:** Dong Peng, Xiao-Yu Liu, Yu-Xi Cheng, Wei Tao, Yong Cheng

**Affiliations:** Department of Gastrointestinal Surgery, The First Affiliated Hospital of Chongqing Medical University, Chongqing, China

**Keywords:** colorectal cancer, surgery, remission, overall survival, type 2 diabetes mellitus

## Abstract

**Purpose:**

The purpose of the current study was to evaluate the impact of colorectal cancer (CRC) surgery on type 2 diabetes mellitus (T2DM) and to analyze the change in T2DM on overall survival after CRC surgery.

**Methods:**

Patients who underwent CRC surgery were retrospectively enrolled from January 2013 to December 2019. The status of T2DM pre- and 1-year after CRC surgery was recorded, and predictive factors for T2DM remission and overall survival were analyzed.

**Results:**

A total of 296 patients were included in this study. Thirty-eight patients experienced remission of T2DM 1 year after CRC surgery, and the remission rate was 12.8%. Weight loss was significantly higher in the T2DM remission group (p = 0.038), and the T2DM duration was significantly shorter in the T2DM remission group (p = 0.015). In the multivariate logistic regression analysis, higher weight loss (p = 0.046, odds ratio = 1.060, 95% CI = 1.001–1.122) and shorter T2DM duration (p = 0.019, odds ratio = 1007, 95% CI = 1.001–1.014) were predictive factors for remission of T2DM. Furthermore, in multivariate Cox regression analysis, lower TNM stage (p = 0.000, odds ratio = 2.147, 95% CI = 1.474–3.128) and T2DM remission (p = 0.033, odds ratio = 2.999, 95% CI = 1.091–8.243) were the predictive factors for better overall survival.

**Conclusion:**

Patients with concurrent CRC and T2DM had a 12.8% remission 1 year after CRC surgery. Higher weight loss and shorter T2DM duration contributed to T2DM remission, and patients with T2DM remission could improve in terms of their overall survival.

## Introduction

Colorectal cancer (CRC) is the third most common cancer and the second leading cause of cancer-related death in the world (1). The 5-year overall survival rates of colon cancer and rectal cancer are approximately 60 and 55%, respectively, in nonmetastatic CRC (2), and surgery is currently the most effective treatment for CRC (3).

The prevalence of type 2 diabetes mellitus (T2DM) is rapidly increasing worldwide (4). Currently, 400 million people suffer from T2DM, and it is estimated that 650 million cases of T2DM will be diagnosed by 2040 (5). It is considered to be one of the most challenging public health problems and greatly affects life expectancy (6). A national survey conducted in 2010 stated that the incidence of T2DM had risen to 11.6%, of which only 25.8% had received conventional treatment, and only 39.7% was well controlled in China (7).

Previous studies reported that T2DM could improve the incidence of CRC (8, 9), and mortality and complications would increase when patients with concurrent CRC and T2DM underwent CRC surgery (10). Furthermore, T2DM has been shown to have significant effects on chemoresistance, and pre-existing T2DM patients decreases overall survival and increases the risk of relapse survival after CRC surgery as well (11, 12).

As previously described, gastric cancer surgery could contribute to T2DM remission (13), and the remission rate was 20–50% (14–16). Moreover, recovery from pre-existing T2DM after radical gastrectomy was associated with better overall survival (14). Similarly, CRC surgery is a digest surgery, however, no previous studies have reported the effect of CRC surgery on T2DM. Therefore, the purpose of this study was to evaluate the impact of CRC surgery on T2DM, and to analyze the change in T2DM on overall survival after CRC surgery.

## Materials and Methods

### Patients

Patients who underwent CRC surgery were retrospectively enrolled from January 2013 to December 2019. Ethical approval from the institutional review board was obtained (2021-046), and informed consent was acquired from all patients.

The inclusion criteria were as follows: 1. patients who were pathologically diagnosed with CRC; 2. patients who underwent radical CRC surgery; and 3. patients diagnosed with concurrent T2DM. The exclusion criteria were as follows: 1. patients who underwent combined organ resection (n = 7); 2. patients with preoperative chemotherapy (n = 11); 3. patients with other endocrine disorders, such as thyroid or adrenal disease (n = 18); 4. Patients who died or were lost to follow-up within one year (n = 17); and 5. patients with incomplete medical records before surgery (n = 43).

**Figure 1:**
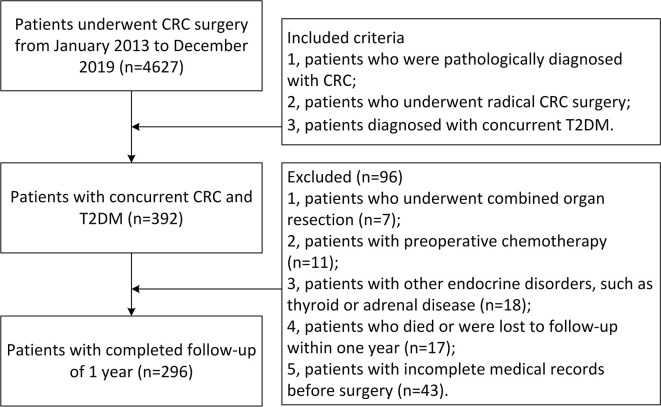
Inclusion and exclusion criteria of patients with concurrent CRC and T2DM. CRC, colorectal cancer; T2DM, type 2 diabetes mellitus.

A total of 4,627 patients were identified in the database, and according to the inclusion criteria, 392 patients with concurrent CRC and T2DM remained. Finally, 296 patients with completed follow-up of 1 year were involved in this study according to the exclusion criteria (**Figure 1**).

### Definitions and Follow-Up

In this study, we analyzed the status of T2DM in pre- and 1-year after CRC surgery. The remission of T2DM was evaluated following American Diabetes Association criteria (17). The T2DM remission group was defined as follows: a return to a normal fasting blood glucose (FBG) range with no requirement for medication or an improved FBG level with reduced medication. The T2DM no remission group was defined as follows: no changes in medication, or aggravation in FBG levels and more medication requirements after CRC surgery (18).

The tumor node metastasis (TNM) stage of CRC was determined according to the 8th edition of the Japanese Classification of Colorectal Carcinoma (19). Radical CRC surgery was conducted, and pathological examinations confirmed R0 resection.

Weight loss referred to the postoperative weight minus preoperative weight, and the duration of T2DM was defined from the first diagnosis of T2DM to CRC surgery. Long-term survival was defined as the time from CRC surgery to death or the end of the study if the patient was still alive. In addition, patients were followed up every three months for the first two years and every six months thereafter to assess tumor recurrence. Carcinoembryonic antigen, abdominal sonogram, chest radiography, colonoscopy and abdominal computerized tomography were arranged if needed.

### Data Collection

The perioperative information was collected from the medical database, and follow-up data were collected from the outpatient service system and through telephone interviews. The extracted clinical data included sex, age, comorbid hypertension, smoking, drinking, weight, body mass index (BMI), T2DM duration, T2DM medication dosage, FBG, tumor site, surgical methods and TNM stage.

### Statistical Analysis

Continuous variables are expressed as the mean ± SD, and categorical variables are expressed as n (%). Chi-square tests and independent-samples t tests were used to compare the differences between the remission group and the no remission group. Univariate and multivariate logistic regression analyses were used to identify predictors of T2DM remission, and univariate and multivariate Cox regression analyses were performed to identify predictive factors for overall survival. Data were analyzed using SPSS (version 20.0) statistical software. A bilateral p value of < 0.05 was considered statistically significant.

## Results

### Baseline Characteristics of Patients With Concurrent CRC and T2DM

A total of 296 patients who had concurrent CRC and T2DM were included in this study, and the glycemic status in the studied patients was stable before and 1 year after CRC surgery. There were 169 males and 127 females, and the average age was 67.8 ± 8.8 years. A total of 163 patients had concurrent CRC, T2DM and hypertension. The preoperative BMI, weight, duration of T2DM, tumor site, smoking, drinking, TNM stage and antidiabetes therapy are shown in **Table 1**.

**Table 1:** Baseline characteristics of patients with concurrent CRC and T2DM.

Characteristics	No. 296
Sex	
Male	169 (57.1%)
Female	127 (43.9%)
Age (mean ± SD), year	67.8 ± 8.8
Hypertension	163 (55.1%)
Smoking	102 (34.5%)
Drinking	110 (37.2%)
BMI preoperative (mean ± SD), kg/m^2^	23.8 ± 3.3
Weight preoperative (mean ± SD), kg	62.3 ± 10.4
Duration of T2DM (mean ± SD), month	80.8 ± 79.3
Tumor site	
Colon	129 (43.6%)
Rectum	167 (56.4%)
TNM stage	
I	58 (19.6%)
II	142 (48.0%)
III	96 (32.4%)
Antidiabetes	
Oral hypoglycemic agent only	252 (85.1%)
Insulin (with or without oral hypoglycemic agent)	44 (14.9%)

Variables are expressed as the mean ± SD or n (%).

CRC, colorectal cancer; T2DM, type 2 diabetes mellitus; BMI, body mass index; TNM, Tumor Node Metastasis.

### Difference Between Remission and No Remission

Thirty-eight patients had remission of T2DM 1 year after CRC surgery, and the remission rate was 12.8%. The difference was analyzed between the remission and no remission groups. Weight loss was -3.0 ± 4.8 kg in the remission group, which was significantly lower than −0.9 ± 5.9 kg in the no remission group (p = 0.038), and T2DM duration was 51.6 ± 77.5 months in the remission group, which was significantly lower than 85.1 ± 78.8 months in the no remission group as well (p = 0.015). However, no difference was found in terms of age, sex, BMI, weight preoperatively, comorbid hypertension, smoking, drinking, tumor site, surgical methods or TNM stage (p >0.05) (**Table 2**).

**Table 2:** Difference between remission and no remission group.

	Remission (38)	No remission (258)	P value
Age (mean ± SD), year	68.5 ± 10.2	67.7 ± 8.6	0.594
Sex			0.647
Male	23	146	
Female	15	112	
BMI (mean ± SD), kg/m^2^	23.6 ± 3.7	23.8 ± 3.0	0.992
Weight preoperative (mean ± SD), kg	62.4 ± 9.7	62.3 ± 10.4	0.950
Weight loss (mean ± SD), kg	−3.0 ± 4.8	−0.9 ± 5.9	0.038*
T2DM duration	51.6 ± 77.5	85.1 ± 78.8	0.015*
Oral hypoglycemic agent only	4	34	0.625
Comorbid hypertension	21	142	0.979
Smoking	14	88	0.719
Drinking	13	97	0.723
Tumor site			0.110
Colon	12	117	
Rectum	26	141	
Surgical methods			0.846
Open	6	44	
Laparoscopic	32	214	
TNM stage			0.376
I	7	51	
II	22	120	
III	9	87	

Variables are expressed as the mean ± SD or n (%), *P-value < 0.05.

BMI, body mass index, T2DM, type 2diabetes mellitus; TNM, Tumor Node Metastasis.

### Univariate and Multivariate Analysis of Predictive Factors for Remission of T2DM

Univariate and multivariate logistic regression analyses were performed to identify predictive factors for remission of T2DM. In univariate analyses, weight loss (p = 0.041, odds ratio = 1.061, 95% CI = 1.003–1.123) and T2DM duration (p = 0.017, odds ratio = 1.007, 95% CI = 1.001–1.013) were predictive factors. Furthermore, in the multivariate analysis, higher weight loss (p = 0.046, odds ratio = 1.060, 95% CI = 1.001–1.122) and shorter T2DM duration (p = 0.019, odds ratio = 1007, 95% CI = 1.001–1.014) were the predictive factors for remission of T2DM (**Table 3**).

**Table 3:** Univariate and multivariate analysis of predictive factors for remission of T2DM.

Risk factors	Univariate analysis		Multivariate analysis	
	OR (95% CI)	P value	OR (95% CI)	P value
Age	0.989 (0.951–1.029)	0.593		
Sex	1.176 (0.587–2.358)	0.647		
BMI	0.999 (0.901–1.109)	0.992		
Weight	0.999 (0.967–1.032)	0.950		
Weight loss	1.061 (1.003–1.123)	0.041*	1.060 (1.001–1.122)	0.046*
Smoking	0.887 (0.437–1.801)	0.741		
Drinking	1.159 (0.566–2.371)	0.687		
Hypertension	0.991 (0.500–1.966)	0.979		
T2DM duration	1.007 (1.001–1.013)	0.017*	1.007 (1.001–1.014)	0.019*
Tumor site	1.798 (0.869–3.718)	0.114		
TMN stage	1.187 (0.736–1.914)	0.482		
Antidiabetes medicine	0.641 (0.216–1.906)	0.424		

*P-value < 0.05.

OR, odds ratio; CI, confidence interval; BMI, body mass index; T2DM, type 2 diabetes mellitus; TNM, Tumor Node Metastasis.

### Univariate and Multivariate Analysis of Overall Survival

**Figure 2:**
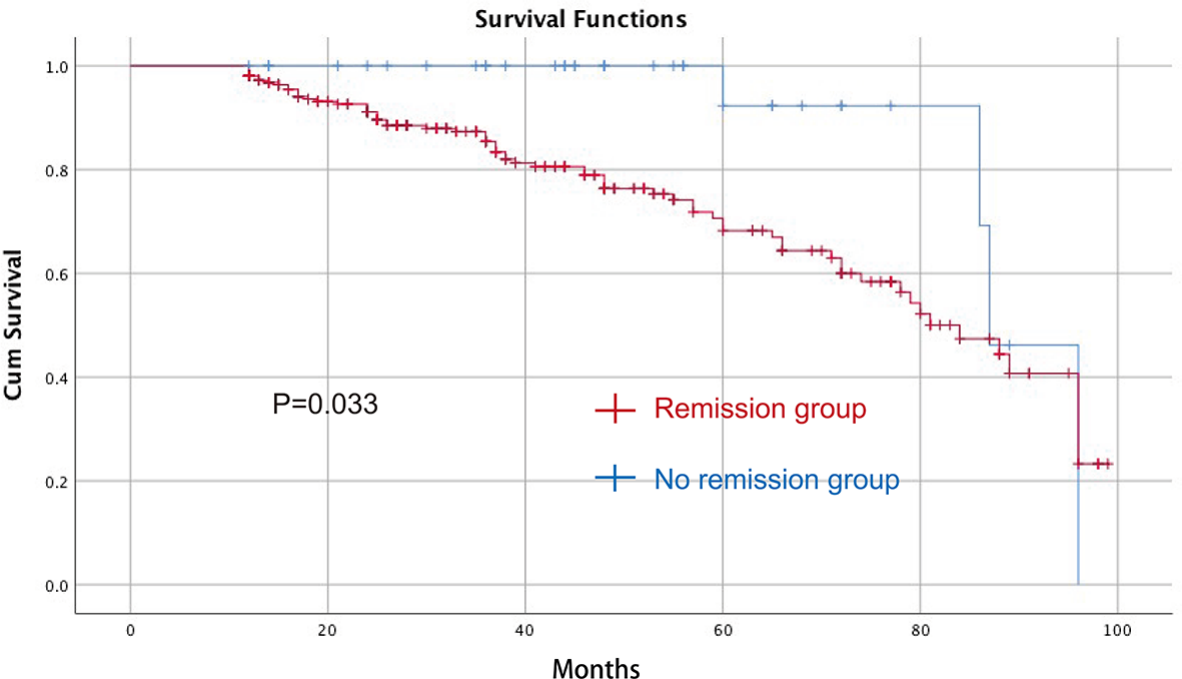
The Kaplan–Meier curve between the T2DM remission group and the no remission group after CRC surgery for OS. CRC, colorectal cancer; T2DM, type 2 diabetes mellitus; OS, overall survival.

The median follow-up time was 37 (12–99) months. Univariate and multivariate Cox regression analyses were performed to identify predictive factors for overall survival. In univariate analyses, TNM stage (p = 0.000, odds ratio = 2.121, 95% CI = 1.468–3.046) and T2DM remission (p = 0.033, odds ratio = 3.000, 95% CI = 1.093–8.237) were predictive factors. In the multivariate analysis, lower TNM stage (p = 0.000, odds ratio = 2.147, 95% CI = 1.474–3.128) and T2DM remission (p = 0.033, odds ratio = 2.999, 95% CI = 1.091–8.243) were predictive factors for better overall survival (**Table 4**). The Kaplan–Meier curve between the T2DM remission group and the no remission group is shown in **Figure 2**.

**Table 4:** Univariate and multivariate analysis of overall survival.

Risk factors	Univariate analysis		Multivariate analysis	
	HR (95% CI)	P value	HR (95% CI)	P value
Age (>/≤68, years)	1.178 (0.736–1.887)	0.495		
Sex (male/female)	1.260 (0.784–2.026)	0.339		
BMI (>/≤23.4)	1.165 (0.726–1.870)	0.527		
Weight (>/≤61.5, kg)	1.048 (0.652–1.685)	0.845		
Weight loss (>/≤−1, kg)	1.031 (0.644–1.649)	0.900		
Hypertension (yes/no)	1.571 (0.965–2.558)	0.069		
T2DM remission (yes/no)	3.000 (1.093–8.237)	0.033*	2.999 (1.091–8.243)	0.033*
T2DM duration (>/≤60, months)	1.455 (0.909–2.331)	0.118		
Tumor site (colon/rectum)	0.621 (0.379–1.017)	0.059		
TMN stage (I/II/III)	2.121 (1.468–3.046)	0.000*	2.147 (1.474–3.128)	0.000*
Smoking (yes/no)	0.735 (0.446–1.210)	0.226		
Drinking (yes/no)	0.954 (0.588–1.548)	0.848		
Antidiabetes medicine (Oral hypoglycemic agent/Insulin)	1.435 (0.686–3.000)	0.337		

*P-value < 0.05.

OR, odds ratio; CI, confidence interval; BMI, body mass index; T2DM, type 2 diabetes mellitus; TNM, Tumor Node Metastasis.

## Discussion

In this study, we analyzed 296 patients who underwent CRC surgery. Thirty-eight patients had remission of T2DM 1 year after CRC surgery, and the remission rate was 12.8%. Weight loss was significantly higher in the T2DM remission group, and T2DM duration was significantly shorter in the T2DM remission group. In multivariate logistic regression analysis, higher weight loss and shorter T2DM duration were the predictive factors for remission of T2DM. Furthermore, in multivariate Cox regression analysis, lower TNM stage and T2DM remission were predictive factors for better overall survival.

As previously described, patients with T2DM have a higher rate of developing CRC (8, 9). It is troublesome for clinicians when performing CRC surgery on patients with T2DM because patients with concurrent CRC and T2DM have greater complications, including infection and anemia, and postoperative mortality increases (10). Better control of T2DM before and after CRC surgery would bring survival benefits for patients (11).

For patients with T2DM, lifestyle changes might also benefit T2DM remission. Patients adhering to low-carbohydrate diets and low-energy diets could experience T2DM remission (20). Increased physical activities could be a predictor for T2DM remission (21). The remission of T2DM reduced the burden of life and the dosage of antidiabetes drugs. Furthermore, the remission of T2DM could reduce DM-related diseases, including diabetic nephropathy, eye disease and peripheral neuropathy (22).

In bariatric surgery, patients had T2DM improvement and apparently lower microvascular and macrovascular disease and mortality after surgery (23). Similarly, patients had remission of T2DM and hypertension after gastric cancer surgery (14, 24), and onco-metabolic surgery occurred. There were some reasons and theories accounting for the remission of T2DM. The foregut theory states that patients undergoing duodenal bypass experience antidiabetic effects (25), whereas the hindgut theory holds that early contact of unabsorbed nutrients with the distal intestine improves T2DM (26). Furthermore, hormones, including ghrelin, GLP-1 and GLP-2, could influence T2DM remission (27).

In this study, patients with a shorter duration of T2DM and greater weight loss had a higher rate of T2DM remission. The possible factor was that a shorter duration of T2DM represented a lower severity of T2DM, and patients with a shorter duration could easily achieve T2DM remission. Higher weight loss is another predictive factor for T2DM remission, and weight loss may be related to lifestyle changes that affected changes of T2DM status (28). However, surgical methods did not have an impact on T2DM remission; therefore, lifestyle change was indicated to be an important factor in T2DM remission (29). Patients might benefit from dietary adjustments, sodium restriction, more exercise and lower alcohol consumption; therefore, T2DM might improve when the lifestyle of patients is modified after CRC surgery.

Pre-existing T2DM patients have a higher rate of developing other second primary malignancies (30), and a worse overall survival after CRC surgery (11, 30). A meta-analysis also reported decreased survival and increased risk of relapse in patients with concurrent CRC and T2DM (11). In the current study, patients with T2DM remission improved in terms of their overall survival after CRC surgery, and T2DM remission improved overall survival in gastric cancer patients (14). The mechanism of improved overall survival remains unclear and might be related to the upregulation of AMP-activated protein kinase, which causes cell cycle arrest in the Gap 1-S (G1-S) phase and inhibits the mTOR pathway (12). Furthermore, modification of lifestyle, dietary restrictions and physical activity are measures for reducing mortality (11).

There are some limitations of this study as well. First, it is a single-center retrospective study, with a total of 296 patients, which is relatively small. Second, postoperative lifestyles, including diet changes and activities, were not included in this study; however, these changes in lifestyle may have influenced T2DM remission. Third, the status of T2DM may change after more than one year, and the specific time of T2DM remission was not documented. Longer follow-up of T2DM change is needed in the future. Finally, we only included patients who survived more than one year in the overall survival analysis, which may have resulted in selection bias, and recurrence-free survival was not documented. Therefore, multicenter and large-sample RCTs concerning the lifestyle changes after CRC surgery are needed in future experiment.

In conclusion, patients with concurrent CRC and T2DM had a 12.8% remission 1-year after CRC surgery. Higher weight loss and shorter T2DM duration contributed to T2DM remission, and patients with T2DM remission could improve their overall survival.

## Data Availability Statement

The original contributions presented in the study are included in the article/supplementary material. Further inquiries can be directed to the corresponding author.

## Ethics Statement

Ethical approval from the institutional review board was obtained (2021-046). The patients/participants provided their written informed consent to participate in this study. Written informed consent was obtained from the individual(s) for the publication of any potentially identifiable images or data included in this article.

## Author Contributions

All authors listed have made a substantial, direct, and intellectual contribution to the work, and approved it for publication.

## Conflict of Interest

The authors declare that the research was conducted in the absence of any commercial or financial relationships that could be construed as a potential conflict of interest.
